# Hypomanic shift observed during rTMS treatment of patients with unipolar depressive disorder: four case reports

**DOI:** 10.1186/1744-859X-12-12

**Published:** 2013-04-24

**Authors:** Eylem Ozten, Gokben Hizli Sayar, Oguz Karamustafalioglu

**Affiliations:** 1 NPIstanbul Hospital, Uskudar University, Alemdag Cad. Siteyolu Sk. No:27, Istanbul, Umraniye 34675, Turkey

**Keywords:** Transcranial magnetic stimulation, Depression, Hypomania

## Abstract

**Objective:**

Repetitive transcranial magnetic stimulation (rTMS) can enhance the excitement of the brain through adjusting the biological activities of the cerebral cortex and has wide biological effects, making it one basic mechanism of therapy for depression. In the treatment of unipolar depressive disorder, almost in every treatment method, hypomanic and manic shifts can be observed. There is still a lack of data regarding manic and hypomanic symptoms triggered by rTMS applications.

**Method:**

We describe four cases with unipolar depression in which high-frequency rTMS over the left dorsolateral prefrontal cortex applied as an add-on antidepressive strategy may have induced a hypomanic episode.

**Results:**

In these cases, 25 Hz rTMS combined with antidepressants may have contributed to the occurrence of hypomanic symptoms.

**Conclusion:**

Using an intensive methodology of rTMS may induce hypomanic or manic symptoms.

## Introduction

Repetitive transcranial magnetic stimulation (rTMS) can increase the excitability of the brain through changing the activity of the cerebral cortex. This has widespread biological effects, which have made it a basic therapeutic mechanism for depression [1]. In 2008, the US Food and Drug Administration approved rTMS as an alternative form of treatment for treatment-resistant depression [2].

Mania has been observed in depression treatments based on pharmacotherapy, electroconvulsive therapy, vagus nerve stimulation, sleep deprivation, and deep brain stimulation used to treat obsessive-compulsive disorder (OCD) [3-6]. In the treatment of unipolar depressive disorders, hypomania and mania have been observed in almost every treatment method, while in the treatment of bipolar disorder depressive episodes, the frequency of hypomania and mania is even higher [7].

Xia et al. reviewed cases of hypomania and mania emerging from rTMS treatments reported during the period 1966 to 2006 [8]. Treatment-emergent hypomania/mania rates were shown to be 0.84% in the active rTMS group and 0.73% in the sham group. This difference was not statistically significant. The same authors showed that during rTMS treatment for unipolar depression, the emergent hypomania/mania rate was 0.34%, while the rate in bipolar disorder depressive episodes, it was 3.1%.

Sakkas et al. looked at treatment-resistant unipolar depression [9]. Manic symptoms were detected with high-frequency 20 Hz rTMS applied over the left dorsolateral prefrontal cortex (LDLPFC), at 1,600 pulses, twice daily and citalopram. Ella et al. reported manic symptoms in a patient with recurrent major depressive disorder treated with 1 Hz rTMS applied over the right dorsolateral prefrontal cortex (RDLPFC) at 1,200 pulses/day and tranylcypromine, haloperidol, and lorazepam [10]. George et al. monitored hypomanic symptoms in a patient with major depressive disorder following a 9-day application of 20-Hz rTMS over the LDLPFC at 400 pulses twice daily [11]. In light of this earlier literature, this article reports the results of the treatment of four patients with unipolar depression who were monitored for symptoms of hypomania/mania following daily rTMS treatment at 25 Hz, 100% motor threshold (MT), and 1,000 pulses/day applied over the LDLPFC.

## Case presentation

### First case

The first case was that of a 21-year-old male patient experiencing his second depressive episode. Three years ago, during his first episode, he benefited from treatment with fluoxetine 20 mg/day. The second episode had begun 4 months earlier. His symptoms included sadness, unhappiness, reluctance, lack of academic progress, social isolation, and difficulty in falling asleep. He had no psychotic symptoms. His Hamilton Depression Rating (HAM-D 17) score on admission was 27, despite treatment with fluoxetine 40 mg/day for the previous 3 months. His grandfather had a history of psychotic disorders. Biochemistry, complete blood count, TIT, TFT, vitamin B_12_, and folic acid levels were normal, and no pathology was detected in electroencephalogram (EEG) and cranial magnetic resonance imaging (MRI). Considered treatment-resistant, escitalopram 10 mg/day and 25 Hz, 1,000 pulses, 100% MT, LDLPFC rTMS were started. After the ninth session, the quantity and speed of his speech had increased, his need for sleep had reduced, and he showed increased mobility, elevated affect, and increased psychomotor activity. His score on the Young Mania Scale was 24. In response to these hypomanic symptoms, the rTMS protocol was changed to 1 Hz, 250 pulses, RDLPFC. Eleven sessions followed using this protocol. Escitalopram was stopped, and the treatment continued with sodium valproate 1,000 mg/day. He was discharged in euthymic condition. In the 12th month of treatment, he was still euthymic, and treatment with sodium valproate was ongoing.

### Second case

The second case was that of a 48-year-old female patient experiencing her second depressive episode. Ten years ago, she recovered from her first depressive episode without medication and with psychotherapy. In the 5 months before treatment, she had complained of symptoms such as anxiety, depressed mood, decreased appetite, weight loss, and anhedonia. The patient had been taking sertraline 200 mg/day for the previous 2 months but showed no improvement. She had no psychotic symptoms. Her HAM-D 17 score was 24. Her biochemical measures were within normal range, and no pathology was detected in EEG and cranial MRI. Considered treatment-resistant, venlafaxine 150 mg/day and 25 Hz, 1,000 pulses, 100% MT, LDLPFC rTMS were started. After 14 sessions, the patient manifested euphoria, sleeplessness, and increased energy and speech. Her score on the Young Mania Scale was 28. In response to these hypomanic symptoms, the rTMS treatment was changed to 1 Hz, 250 pulses, RDLPFC, and six sessions were applied. Venlafaxine was stopped, and the treatment was continued with sodium valproate 1,000 mg/day. She was discharged in euthymic condition. By the ninth month of treatment, she was still euthymic, and sodium valproate treatment was ongoing.

### Third case

The third case concerned a 38-year-old female patient diagnosed with unipolar depressive disorder. Although she had been taking paroxetine 40 mg/day for the past 3 years, she had not taken any psychotropic medications in the previous 2 years. She described a ten-month depressive episode and had been taking fluoxetine 40 mg/day for the previous 2 months with no improvement. Her symptoms included depression, anhedonia, anxiety, low self-esteem, and decreased functioning. Her HAM-D 17 score was 22. No OCD symptoms were observed. Her biochemical measures were within normal range, and no pathology was detected in EEG and cranial MRI. Considered to be suffering from treatment-resistant depression, her fluoxetine dose was adjusted to 60 mg/day, and 25 Hz, 1,000 pulses, 100% MT, LDLPFC rTMS was started. No psychotic symptoms were detected. After 12 sessions, she manifested euphoria, sleeplessness, and increased energy and speech, and her score on the Young Mania Scale was 20. In response to her hypomanic symptoms, rTMS was changed to 1 Hz, 250 pulses, RDLPFC, and eight sessions were applied. Fluoxetine was stopped, and treatment continued with sodium valproate 1,000 mg/day. She was discharged in euthymic condition after 2 weeks. In the fourth month of treatment, she was still euthymic, and treatment with sodium valproate was ongoing.

### Fourth case

This concerned a 23-year-old female patient experiencing her second depressive episode. During her first episode 3 years earlier, she had attempted suicide. Consequently, she was hospitalized, and drug therapy consisted of escitalopram 20 mg/day and trazadone 100 mg/day. During follow-up, she was considered euthymic. Her treatment had gradually stopped working 1 year earlier, and 3 months earlier, her depressive symptoms including difficulty in falling asleep, anhedonia, lack of drive, loss of appetite, weight loss, low self-esteem, and loss of functionality had returned. She had no psychotic symptoms. Her HAM-D 17 score was 26. Her biochemical measures were within normal range, and no pathology was detected in EEG. As the patient had previously benefited from treatment with escitalopram and trazadone, this was resumed but with no improvement. Drug therapy was changed to escitalopram 20 mg/day and mirtazapine 30 mg/day, and 25 Hz, 1,000 pulses, 100% MT, LDLPFC rTMS was added to the treatment program. After 12 sessions, she manifested euphoria, sleeplessness, and increased energy and speech. Her Young Mania score was 22. In response to these hypomanic symptoms, 1 Hz, 250 pulses, RDLPFC rTMS was applied for eight sessions. Escitalopram and mirtazapine were stopped, and the treatment was continued with sodium valproate 1,000 mg/day. She was discharged in euthymic condition. In the eighth month of treatment, she was still euthymic, and her sodium valproate treatment was ongoing. Table 1 summarizes these four cases.

**Table 1:** Summary of cases

**Case**	**HAM-D score**	**Medication during rTMS**	**Psychiatric family history**	**History of bipolarity**	**TMS application procedure**	**Session when hypomania was observed**	**Hypomania treatment**
I	27	Escitalopram 10 mg/day	Grandfather had a psychotic disorder	None	25 Hz, 1,000 pulses, LDLPFC, 100% MT, one session daily	9	Escitalopram stopped. Valproic acid added. Eleven sessions of 1 Hz, 250 pulses, RDLPFC rTMS applied
II	24	Venlafaxine 150 mg/day	None	None	25 Hz, 1,000 pulses, LDLPFC, 100% MT, one session daily	14	Venlafaxine stopped. Valproic acid added. Six sessions of 1 Hz, 250 pulses, RDLPFC rTMS applied
III	22	Fluoxetine 60 mg/day	None	None	25 Hz, 1,000 pulses, LDLPFC, 100% MT, one session daily	12	Fluoxetine stopped. Valproic acid added. Eight sessions of 1 Hz, 250 pulses, RDLPFC rTMS applied
IV	26	Escitalopram 20 mg/day, mirtazapine 30 mg/day	None	None	25 Hz, 1000 pulses, LDLPFC, 1,00% MT, one session daily	12	Escitalopram and mirtazapine stopped. Valproic acid added. Eight sessions of 1 Hz, 250 pulses, RDLPFC rTMS applied

## Discussion

Bipolar depression is associated with mania-hypomania, and natural switch rates are considered to be approximately 4.6% to 6.7% [8]. The review by Xia et al. of controlled clinical studies showed that the risk of hypomania-mania shift in bipolar depression was nine times higher than in unipolar depression [8]. They argued that this might be because patients with bipolar depression had been mistakenly evaluated as unipolar. In order to avoid such mistakes, we investigated both the manic and hypomanic histories of patients and their families in detail, and no such history was found.

The same study found that emergent hypomania-mania rates in rTMS treatments were 0.84% in the rTMS group and 0.73% in the sham group and that following the application of rTMS treatment for unipolar depression, the hypomania-mania shift rate was 0.34%, while in bipolar disorder depressive phase, the rate was 3.1%. Mood switching was considered to be related to the nature of the disease and aggressive doses of rTMS [8]. Similarly, in the case studies reported by Sakkas et al. and Hausmann et al., hypomania-mania symptoms were observed during 20 Hz rTMS applications [9,12]. In the present report, cases of 419 patients treated between January 2007 and December 2011 were evaluated.

In the Hausmann et al. study, manic symptoms occurred during 20 Hz, 2,000 pulses rTMS treatment of a patient with bipolar depression [12]. When the protocol was changed to 1 Hz, 1,200 pulses, RDLPFC, and antidepressants were replaced by clozapine, manic symptoms disappeared. The rTMS protocol in our four cases was 25 Hz, LDLPFC, 1,000 pulses/day, and 100% MT. Hypomanic symptoms were observed during the initial treatment period in all four of our cases. In each case, antidepressants were replaced by valproic acid, and the protocol was changed to 1 Hz, 250 pulses RDLPFC. In all cases, hypomanic symptoms disappeared following the protocol changes. This could be related to either the impact of the change in medication or changes in the rTMS application. However, it should be noted that Ella et al. observed manic symptoms in a patient with recurrent major depressive disorder taking tranylcypromine, haloperidol, and lorazepam and treated with 1 Hz, 1,200 pulses, RDLPFC rTMS [10]. This result is in contrast to our findings and demonstrates that there is still a lack of data regarding manic and hypomanic symptoms triggered by rTMS applications.

It is known that any remedial effects of treatment can potentially arise from two sources: one related to the specific properties of the treatment and the other associated with the patient's expectations for the treatment (placebo effect). The magnitude of the placebo effect varies according to its supposed effectiveness and emotional impact on the treated subject [13]. Many studies reported response rates for patients who received placebo treatment. Klein et al. reported a control group response rate as high as 25% [14]. Patients receiving placebo rTMS may receive a small dose of magnetic energy that may alter their depression.

There are many variations in the way rTMS can be given as a clinical treatment, involving choices over treatment site, stimulation parameters, and treatment course. Studies to date have differed with respect to these, with almost no two studies using identical rTMS parameters, except in deliberate attempts at replication. High-frequency rTMS to the left prefrontal cortex has been used in most trials, a choice influenced by positive early results from this approach. Imaging studies have shown evidence of reduced blood flow in the left prefrontal cortex in patients with depression [15]. Some investigators have tried low-frequency rTMS to the right prefrontal cortex. Motor cortex studies suggest that high- and low-frequency rTMS have opposite effects on the excitability of neurons in the brain cortex [16]. There is considerable evidence from neuropsychological, lesion, and imaging studies that the left and right hemispheres have contrasting roles in mood regulation [17]. Therefore, it might be expected that low-frequency rTMS to the right prefrontal cortex may be as likely to have antidepressant effects as high-frequency rTMS to the left prefrontal cortex. However, there is no strong evidence for either side in the peer-reviewed literature. Stimulus frequency of rTMS was within the range of 5 to 25 Hz in several studies as changes in cortical excitability have been demonstrated with this range of frequency [16]. Results suggest that higher stimulus frequencies may have greater antidepressant potency. The majority of rTMS trials have reported stimulus intensity relative to the subject's resting motor threshold, that is, the lowest stimulus intensity necessary to produce a motor response in a relaxed contralateral muscle when TMS is given over the primary motor cortex. Researchers have reported that stimulus intensity is an important factor in inducing lasting changes in cortical excitability that may be responsible for antidepressant effects. In depressed subjects, studies comparing different intensities of stimulation (100% and 90% of motor threshold) showed that higher stimulation intensity was significantly associated with greater improvement [18]. However, an overview of studies in the literature does not support an association between higher intensities (110% or 120% of motor threshold) and greater response rates. rTMS trials have moved in the direction of administering longer-duration protocols with greater doses of magnetic pulses [19-21]. These clinical studies suggest that treating depressed patients with higher doses improves response and remission rates with rTMS.

The main limitation of our report is that, in each of the four cases, the rTMS was initiated together with initiation or increase of high-dose antidepressant medication. Therefore, the ‘switch’ may also be due to the antidepressant medication and cannot be necessarily contributed to rTMS. However, further research is needed on this topic.

## Conclusion

We conclude that using an intensive methodology of rTMS may induce hypomanic or manic symptoms.

## Consent

Written informed consent was obtained from the patients for publication of this Case report and any accompanying images. A copy of the written consent is available for review by the Editor-in-Chief of this journal.

## Abbreviations

rTMS: Repetitive transcranial magnetic stimulation; OCD: Obsessive-compulsive disorder; LDLPFC: Left dorsolateral prefrontal cortex; RDLPFC: Right dorsolateral prefrontal cortex; MT: Motor threshold; HAM-D: Hamilton Depression Rating.

## Competing interests

The authors declare that they have no competing interests.

## Authors’ contributions

EO analyzed and interpreted the patient data. GHS performed the HAM-D ratings and wrote the manuscript. OK was a major contributor in writing the manuscript. All authors read and approved the final manuscript.
